# Congenital intrarenal arteriovenous malformation presenting with gross hematuria after endoscopic intervention: a case report

**DOI:** 10.1186/1752-1947-2-326

**Published:** 2008-10-12

**Authors:** Michael Seitz, Tobias Waggershauser, Wael Khoder

**Affiliations:** 1 Department of Urology, University Hospital Grosshadern, Marchioninistrasse, 81377 Munich, Germany; 2 Department of Radiology, University Hospital Grosshadern, Marchioninistrasse, 81377 Munich, Germany

## Abstract

**Introduction:**

Although diagnostic ureterorenoscopy is a minimally invasive and effective diagnostic procedure, it has the potential for significant postoperative complications. We report the first case in the literature of intrarenal arteriovenous fistulas causing hemodynamic effective anemia 4 days after ureterorenoscopic biopsy.

**Case presentation:**

A 63-year-old Caucasian woman presented with hemodynamic effective macrohematuria (hemoglobin 70 g/liter) 4 days after ureterorenoscopy and biopsy of the upper pole collecting system due to recurrent microhematuria. Duplex-sonography and computed tomography angiography revealed multiple arteriovenous fistulas and erosions into the calyceal system. Intra-arterial digital subtraction angiography confirmed this condition. After superselective embolization of the arteriovenous fistulas, the patient had no further episodes of bleeding or microhematuria.

**Conclusion:**

If malignancies, urolithiasis or urinary tract infections are ruled out by common diagnostic procedures as the cause of recurrent minor or gross hematuria, the possibility of arteriovenous fistulas should be included in the differential diagnosis and Duplex-Sonography or the more invasive selective renal arteriography should be performed as this is the most definitive method for diagnosing arteriovenous fistula.

## Introduction

Although arteriovenous fistulas are rare conditions, they have a considerable clinical impact. In fact they may cause hypertension, local thrombosis, peripheral embolization, high output cardiac failure and hematuria. Although ureterorenoscopy is a minimally invasive and effective diagnostic and therapeutic procedure, it has the potential for significant postoperative complications. We report a case of intrarenal arteriovenous fistulas causing hemodynamic effective anemia 4 days after ureterorenoscopic biopsy.

## Case presentation

**Figure 1:**
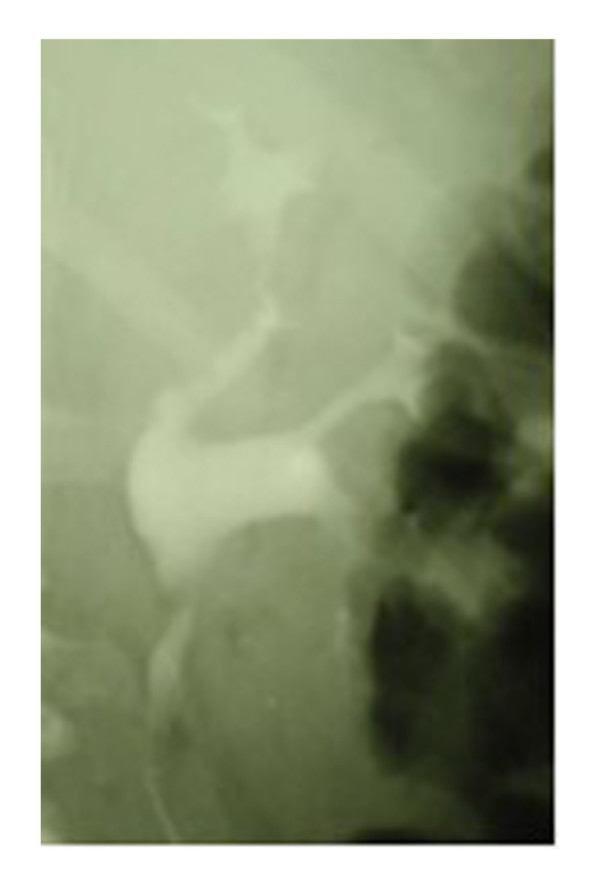
**Retrograde ureteropyelography**. Retrograde ureteropyelography demonstrates an irregular configuration of the upper pole collecting system.

A 63-year-old woman presented with recurrent microhematuria. She had no history of flank pain, macrohematuria, hypertension, renal trauma or percutaneous instrumentation. Physical examination was normal and specifically, there was no abdominal bruit on auscultation. She had a blood pressure of RR 130/80 mmHg. Routine laboratory tests were within normal limits. Urinalysis showed no evidence of infection but was positive for erythrocytes. An initial renal ultrasound revealed a discrete hypoechogeneity of the left upper renal pole. An intravenous pyelogram was performed demonstrating an irregular configuration of the upper pole collecting system, which was also seen in a retrograde ureteropyelography (Figure 1). Cystoscopy as well as ureterorenoscopy (URS) revealed no suspicious formation within the bladder or along the left ureter or in the renal pelvis. Tissue around blood clots in the upper calyceal group was biopsied. Cytology and histology did not identify malignant cells. The patient was discharged with a ureteral stent. Four days after the intervention, emergency admission was necessary due to a hemodynamic effective macrohematuria (hemoglobin 70 g/liter) causing a bladder coagulum, which made transurethral evacuation necessary.

**Figure 2:**
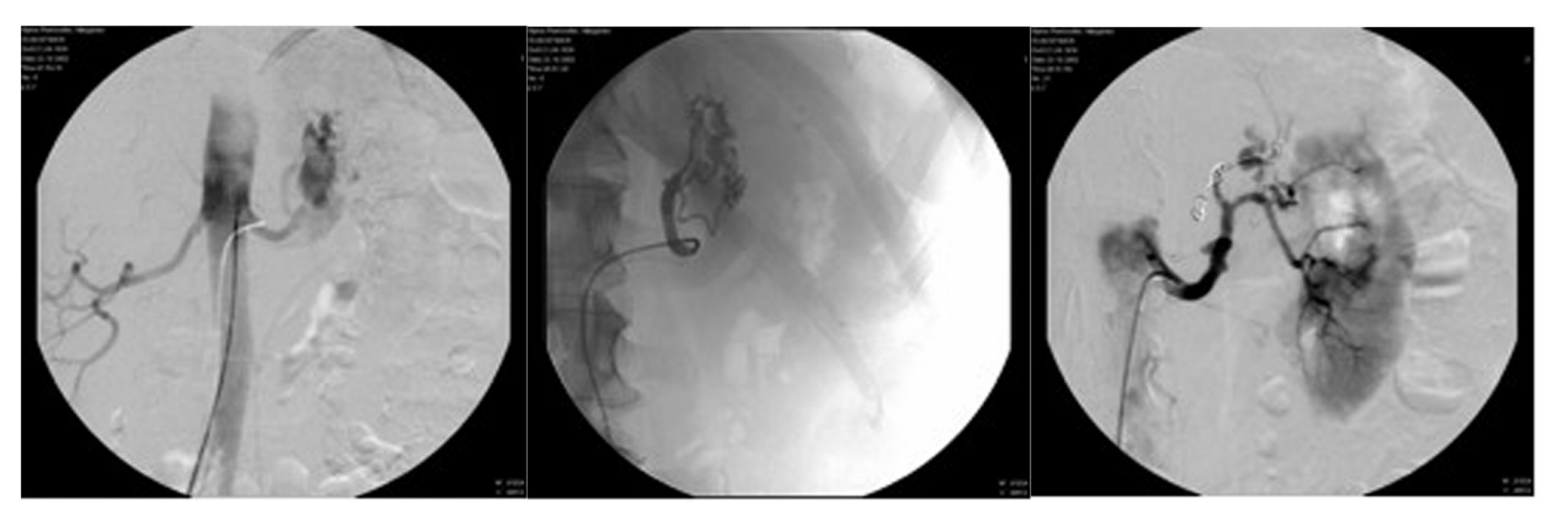
**Angiography**. In the early arterial phase, intra-arterial digital subtraction angiography demonstrates arteriovenous fistulas between a subsegmental branch of the renal artery and the renal vein (left, central) and these were superselectively embolized by 8 Platin-coils with cotton filaments (right). Angiographically, no significant differences are noted in parenchymal perfusion before and after intervention. Pathologic neoplastic vessels are ruled out radiomorphologically.

Duplex-sonography and computed tomography angiography (CTA) were then carried out and revealed multiple arteriovenous fistulas (AVFs) and erosions into the calyceal system. Intra-arterial digital subtraction angiography (i.a. DSA, Figure 2) in the early arterial phase showed arteriovenous fistulas between a subsegmental branch of the renal artery and the renal vein and these were superselectively embolized by 8 Platin-coils with cotton filaments. Angiographically, no significant differences in parenchymal perfusion were noted before and after intervention. Pathologic neoplastic vessels were ruled out radiomorphologically. Five months after intervention, a control computed tomogram showed no recurrent AVF or malignancy. The patient had no further episodes of bleeding or microhematuria.

## Discussion

Arteriovenous fistulas, first described by Varela in 1928, are rare conditions, which, however, have a considerable clinical impact [1]. In fact, they may cause hypertension, local thrombosis, peripheral embolization, high output cardiac failure and hematuria [2]. There are two types of AVF, classified as congenital and acquired [3]. In total, 70 to 80% of all AVFs are of the acquired type and may be secondary to trauma, renal surgery, inflammation, neoplasia or percutaneous needle biopsy, the latter contributing to recent increased incidence. Acquired renal AVFs may be located throughout the whole kidney. Angiographically, they appear as solitary communications between arteries and veins. More than 70% of these fistulas close spontaneously within a few weeks or months without active intervention. Therefore the common strategy in asymptomatic patients with incidental detection of AVFs is to 'wait and watch' [4].

In 20 to 30% of all cases, an arteriovenous fistula is a congenital condition usually located in the upper pole (45% of cases) but may also appear in the midportion or the lower pole of the kidney in equal ratio topographically beneath the calyceal or pelvic mucosa. Congenital AVFs are characterized angiographically, as in our patient, by their cirsoid configuration with multiple communications between arteries (main or segmental renal arteries) and veins [2,4].

Based on the angiographic criteria, a second form of congenital AVF exists which is classified as the aneurysmal type and has been mentioned in the literature as a spontaneous or idiopathic fistula [4]. While the latter predominantly present with cardiovascular symptoms, the cirsoid forms show a high incidence of gross hematuria [2].

In the pericalyceal renal parenchyma, the small interlobular arteries and their corresponding veins as well as existing AVFs are in close proximity to the collecting system. This explains recurrent hematuria in more than 75% of individuals and possible filling defects or reduced function of the affected kidney in the excretory urography, but these are absent in 50% of cases [2]. In our patient, we postulate that, due to the biopsy during endoscopic intervention, a perforation had occurred and venous dilatations of the AVFs eroded into the collecting system causing gross hematuria. Active management was necessary due to hemodynamic effective gross hematuria. Selective renal arteriography, as the most definitive method for diagnosing the lesion, was performed with simultaneous superselective coil embolization. This treatment method is well accepted in such conditions since it avoids surgery. Parenchymal infarction secondary to embolization can be limited to the region which is supplied by the artery containing the lesion. This is especially important in patients with only one functioning kidney or renal insufficiency. The technique is also indicated in patients who are considered poor surgical candidates since the procedure is performed under local anesthesia with low morbidity and low risk of complications [5-7].

In contrast to patients presenting with hematuria, we suggest nephrectomy or partial nephrectomy as the treatment of choice in individuals with symptoms of alterations in the cardiovascular system such as renin-mediated hypertension due to fistula-related relative ischemia or high-output cardiac failure caused by increased venous return.

## Conclusion

Congenital AVFs are rare conditions which may cause cardiovascular complications (in 50% of cases) and recurrent hematuria in more than 75% of individuals.

If malignancies, urolithiasis or urinary tract infections are ruled out by common diagnostic procedures as the cause of recurrent minor or gross hematuria, the possibility of AVFs should be included in the differential diagnosis and Duplex-Sonography, or the more invasive selective renal arteriography, as the most definitive method for diagnosing AVF, should be performed. Depending on the general condition of the patient and their symptoms, the treatments of choice include nephrectomy and partial nephrectomy but most urologists aim for superselective embolization.

## Abbreviations

AVF: arteriovenous fistula; CTA: computed tomography angiography; DSA: digital subtraction angiography; i.a. DSA: intra-arterial digital subtraction angiography; RR: blood pressure (measured by the technique of Riva Rocci); URS: ureterorenoscopy

## Competing interests

The authors declare that they have no competing interests.

## Authors' contributions

MS made substantial contributions to acquisition and interpretation of the data and drafted the manuscript. TW carried out the imaging studies and performed the embolization. WK managed the critically ill patient clinically and also contributed substantially to the interpretation of the literature.

## Consent

Written informed consent was obtained from the patient for publication of this case report and any accompanying images. A copy of the written consent is available for review by the Editor-in-Chief of this journal.
